# Pilot performance of a dedicated prostate PET suitable for diagnosis and biopsy guidance

**DOI:** 10.1186/s40658-020-00305-y

**Published:** 2020-06-05

**Authors:** Gabriel Cañizares, Andrea Gonzalez-Montoro, Marta Freire, Efthymios Lamprou, John Barrio, Filomeno Sanchez, José M. Benlloch, Liczandro Hernandez, Laura Moliner, Luis F. Vidal, Irene Torres, Pablo Sopena, Cesar D. Vera-Donoso, Pilar Bello, Julio Barbera, Antonio J. Gonzalez

**Affiliations:** 1 Instituto de Instrumentación para Imagen Molecular (I3M), Centro Mixto CSIC — Universitat Politècnica de València, Camino de Vera s/n, 46022 Valencia, Spain; 2 Servicio de Medicina Nuclear, Área Clínica de Imagen Médica, Hospital Univ. y Polit. La Fe, 46026 Valencia, Spain; 3 Urology Department, La Fe, 46026 Valencia, Spain; 4 Oncovision, Carrer de Jeroni de Montsoriu, 92, 46022 Valencia, Spain

## Abstract

**Background:**

Prostate cancer (PCa) represents one of the most common types of cancers facing the male population. Nowadays, to confirm PCa, systematic or multiparametric MRI-targeted transrectal or transperineal biopsies of the prostate are required. However, due to the lack of an accurate imaging technique capable to precisely locate cancerous cells in the prostate, ultrasound biopsies sample random parts of the prostate and, therefore, it is possible to miss regions where those cancerous cells are present. In spite of the improvement with multiparametric MRI, the low reproducibility of its reading undermines the specificity of the method. Recent development of prostate-specific radiotracers has grown the interest on using positron emission tomography (PET) scanners for this purpose, but technological improvements are still required (current scanners have resolutions in the range of 4–5 mm).

**Results:**

The main goal of this work is to improve state-of-the-art PCa imaging and diagnosis. We have focused our efforts on the design of a novel prostate-dedicated PET scanner, named ProsPET. This system has small scanner dimensions defined by a ring of just 41 cm inner diameter. In this work, we report the design, implementation, and evaluation (both through simulations and real data) of the ProsPET scanner. We have been able to achieve < 2 mm resolution in reconstructed images and high sensitivity. In addition, we have included a comparison with the Philips Gemini-TF scanner, which is used for routine imaging of PCa patients. The ProsPET exhibits better contrast, especially for rod sizes as small as 4.5 mm in diameter. Finally, we also show the first reconstructed image of a PCa patient acquired with the ProsPET.

**Conclusions:**

We have designed and built a prostate specific PET system, with a small footprint and improved spatial resolution when compared to conventional whole-body PET scanners. The gamma ray impact within each detector block includes accurate DOI determination, correcting for the parallax error. The potential role of combined organ-dedicated prostate-specific membrane antigen (PSMA) PET and ultrasound devices, as a prebiopsy diagnostic tool, could be used to guide sampling of the most aggressive sites in the prostate.

## Introduction

Prostate cancer (PCa) is a major worldwide health concern facing the male population. PCa is the most common type of cancer among men in Europe, which is closely followed by lung and colorectal cancer, with 1,276,106 cases reported in 2018 and causing 358,989 deaths (3.8% of all deaths caused by cancer in men) in 2018 [1]. Although mortality rates are generally high in populations of African descent, intermediate in the USA, and very low in Asia, a relatively less variation is observed in mortality rates worldwide [2]. Otherwise, if we consider future epidemiological previsions, 2,293,818 new cases are estimated until 2040, observing therefore a small variation in mortality (an increase of 1.05%) [3].

The most frequently used method for imaging the prostate is transrectal ultrasound (TRUS). However, less than 60% of tumors—usually advanced tumors—are visible with TRUS [4]. Therefore, in clinical diagnosis, grey-scale TRUS is not reliable at detecting PCa [5]. Thus, there is evidence that US biopsies are useful just in a systematic approach.

At present, multiparametric magnetic resonance imaging (mpMRI) is increasingly used to localize suspicious areas that could be targeted by so-called magnetic resonance imaging-targeted biopsies. However, in a recent meta-analysis which compared mpMRI to template biopsies (> 20 cores) in biopsy-naive and repeat-biopsy settings, mpMRI had a pooled sensitivity of 0.91 (95% CI 0.83–0.95) and a pooled specificity of 0.37 (95% CI 0.29–0.46) for ISUP grade > 2 cancers. For cancers with International Society of Urological Pathology grade > 3, mpMRI pooled sensitivity and specificity were 0.95 (95% CI 0.87–0.99) and 0.35 (95% CI 0.26–0.46), respectively [6]. Perhaps this low specificity is due to the poor mpMRI reproducibility and the reason why the European Guidelines on PCa itself states that “Despite the use of the PIRADSv2 scoring system, mpMRI inter-reader reproducibility remains moderate at best” [7].

Molecular imaging using positron emission tomography (PET) is an alternative technique. However, when combined with fluorodeoxyglucose (FDG), the diagnosis of PCa decreases due to the low and heterogeneous consumption of glucose by PCa [8]. Recent developments of new PET ligands such as ^18^F-labeled choline analogs, ^11^C-acetate, or ^18^F-fluorodihydrotestosterone have shown promising results in the detection of malignant lesions in PCa [9, 10].

A more advanced solution for diagnosing PCa is to search for PCa-specific antigenic targets and to generate agents that are able to specifically bind such as the prostate-specific membrane antigen (PSMA), which is overexpressed in PCa tissue. While advances in conventional imaging will continue, antibody (Ab) and small molecule imaging exemplified by PSMA targeting have the greatest potential to improve diagnostic sensitivity and specificity [11]. State-of-the-art PET scanners present spatial resolutions of 3–5 mm, which is in a sharp mismatch with the sizes of the structures and cancerous lesions that need to be visualized in the prostate and, therefore, these scanners are not able to detect small tumoral lesions [12]. Interestingly, the spatial resolution requirements for a PET, accurate enough for the detection of prostatic lesions, are practically the same as for small animal models (around 1 mm). However, the ultimate limit is imposed by the specificity of the imaging agents and uptake in the lesions, while the PET imager operates at the physical limit of performance [13–15].

To improve state-of-the-art PCa diagnosis, the proposed imaging system must allow one for a precise image-based diagnosis and for accurate biopsy guidance. To meet these requirements, the collaboration with physicians is the key to ensure an optimal determination of the required dimensions of a prostate-dedicated PET scanner, in addition to other parameters such as a small footprint in order to be easily moved between different rooms within the Nuclear Medicine or Radiology departments. In this work, we describe the design and performance evaluation of a dedicated PET system for prostate imaging, with a small geometry both in the number of detectors and the system itself, but with a high performance comparable to whole-body PET scanners.

In the proposed prostate-dedicated scanner, the spatial resolution is almost uniform across the entire field of view (FOV) due to the implementation of the photon depth of interaction (DOI) information, which is one of the advantages of using monolithic scintillation crystals for the detector design. We have carried out an evaluation of the spatial resolution, sensitivity, noise-equivalent count rate (NECR), and image quality of the proposed geometry. Simulations based on Monte Carlo (MC) of the system have also been carried out and accordingly compared to the experimental results. Moreover, a patient was scanned on the prototype with successful results when compared to standard whole-body PET scanners.

## Materials and methods

### Scanner geometry

**Fig. 1:**
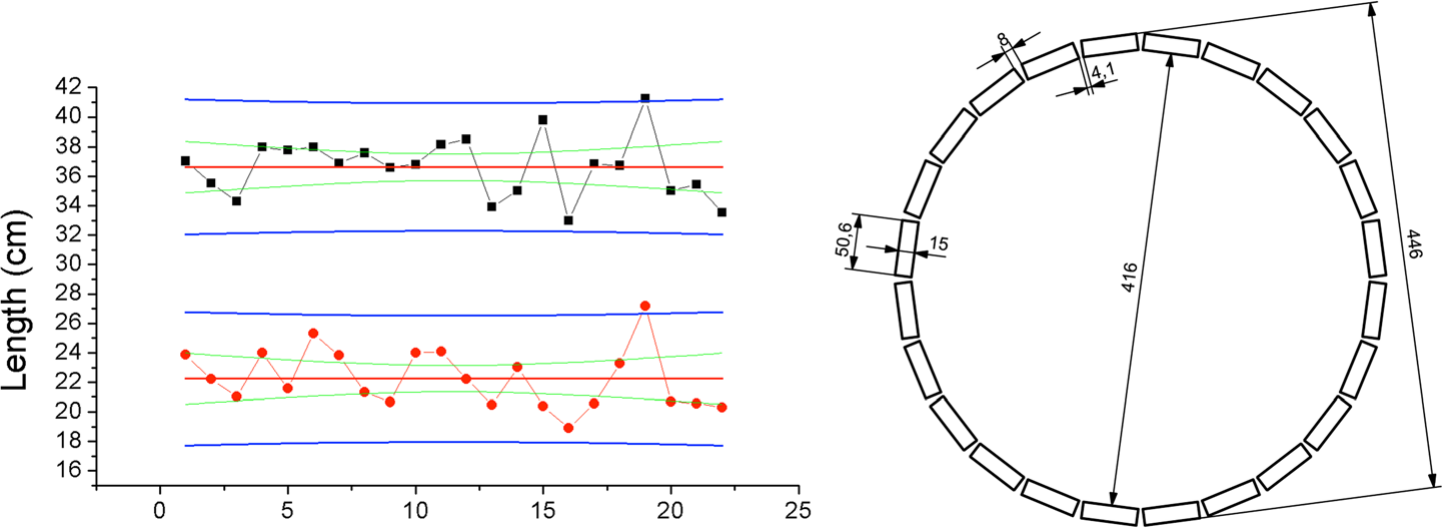
Left, study on patient dimensions. Black squares represent the “wide” measurements, whereas the red circles are the patient thickness. Right, prostate PET ring dimensions (in mm)

Using MRI images from 22 patients, we have determined the average abdominal dimensions at the axial place where the prostate is located. We found a wide size and patient thickness of 36 cm and 22 cm, respectively (see Fig. 1 left). Considering these values, the prostate-dedicated PET ring design, named ProsPET, has been designed with an aperture of about 410 mm in diameter. The ProsPET ring includes 24 detector blocks separated by a thin gap of just 4 mm. Figure 1 right shows a sketch of the ring geometry and detector positions.

### System description

Each one of the 24 detector modules of the ProsPET ring is based on a single monolithic LYSO scintillation crystal of 50 × 50 × 15 mm^3^. All faces have been polished and the four lateral ones (50 × 15 mm^2^) black painted, in order to reduce undesired internal reflections. The entrance face of the scintillation block (50 × 50 mm^2^) was covered with a retroreflector layer that bounces back the scintillation light to the emission point preserving the light distribution profile [16, 17] while increasing the light collection efficiency. The exit face of the scintillator was coupled by means of optical grease (BC630, Saint Gobain) to a photosensor array of 12 × 12 SiPMs. In particular, we made use of silicon photomultiplier (SiPM) arrays of the type C-Series (SensL, now On-Semi) with 3 × 3 mm^2^ active area, 4.2 mm pitch, and 35 microns cell size [18]. A custom readout electronics based on passive components reduces the 144 SiPM signals to only 24. In particular, the 12 SiPM signals of each row and column of the photosensor array were summed and pre-amplified before transferring to the data acquisition system (DAQ). This readout scheme allows one to characterize the scintillation light distributions in monolithic crystals [16]. The PET scanner has been assembled without any forced cooling approach, but simply requiring the room to be at a stable temperature in between 20 and 25°C (variation ± 0.5°C). The DAQ system was installed in a cabinet under the patient’s bed, where the patient is in supine position.

For each detector block, thin multicoaxial cables (SAMTEC) have been used to exchange the 24 (12 + 12 row and columns) analog signals, the trigger signal (sum of all 24), and the temperature sensor, as well as the amplifiers and SiPM bias. The trigger logic has been programmed to digitize signals only when two trigger signals are within a coincidence window of 5 ns. Charge integrators with a window of 250 ns, and 12-bit precision, are used.

Every detector allows coincidences with its 13 opposite detectors. This defines a transaxial and axial FOV of about 300 and 46 mm, respectively. The axial FOV can be increased to about 80 mm by axially displacing the ring and allowing certain image overlapping (multiacquisition process).

**Fig. 2:**
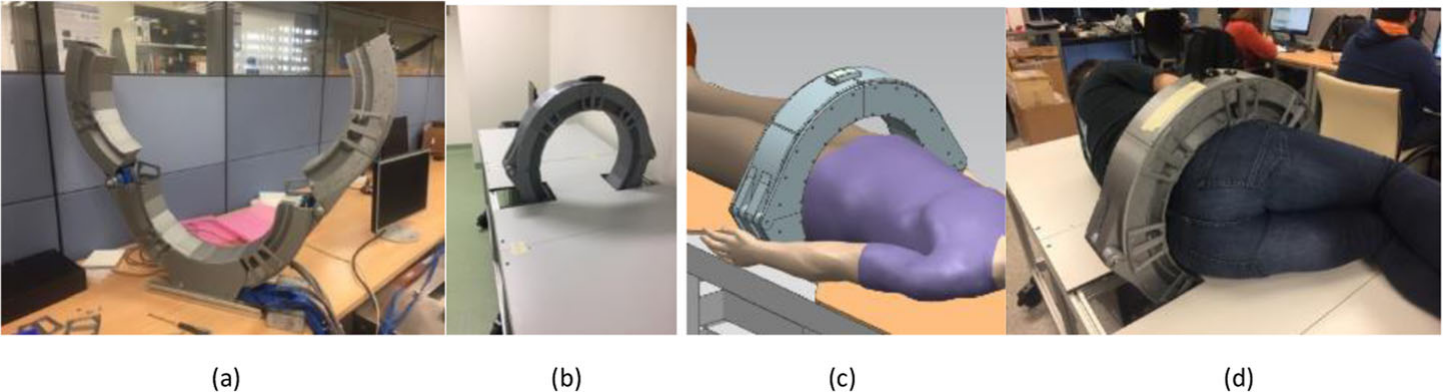
Photographs of the scanner. **a** During assembly showing the movable sections. **b** Installed together with the bed. **c** System representation with a virtual patient. **d** During an ergonomic test showing the possibility for rectal biopsies

In order to facilitate patients to properly position in the bed and into the scanner, the system has two movable parts that open and close with an accuracy of about 0.5 mm. In Fig. 2, from left to right, one can see the detector ring when it is open, installed in the acquisition bed, with a patient in supine position, and an example in the lateral cubit, respectively.

### Calibration data

**Fig. 3:**
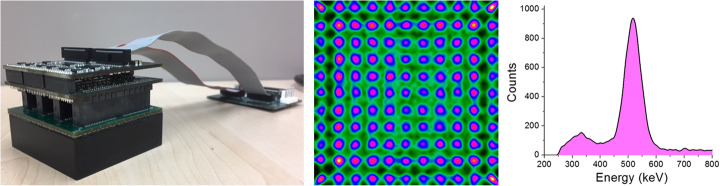
Left, photograph of the detector block, photosensor, and frontend electronics. Center, calibrated flood map of the 11 × 11 sources array used of position calibration. Right, calibrated energy profile of one detector

For every detector block, we run calibration processes of the impact position for both the planar and DOI coordinates, as well as for the energy. This calibration process is either based on 1D polynomials [19] or Voronoi diagrams [20] (see Fig. 3). Similar image performance is obtained with either method. See reference [20] for a detailed comparison of both calibration procedures. Planar coordinates are calculated by raising the 12 digitized signals for each projection to the power of two, before center of gravity (CoG) calculation. The DOI coordinate is estimated for each event as the average for rows and columns (*r*,*c*) of the ratio of the sum of all 12 signals (photon energy, *E*) to its maximum value (퐸/I_max_)_*r*,*c*_, [16]. Independently of the calibration procedure, a continuous DOI correction for each line of response (LOR) is carried out. After calibration, list-mode data files are generated, prior to reconstruction.

### Simulation platform

We have carried out Monte Carlo simulations of the proposed prostate PET system using GATE v7.2 [21]. The code source is Geant4. For the simulations, the already described LYSO scintillator block was modeled and placed in a ring configuration as schematically shown in Fig. 1. It has to be mentioned that, for simplicity, we have defined back-to-back (511 keV) sources instead of positron emitter sources and therefore some variations were expected between the simulation results and the experimental performance of the detectors.

The simulations results have been used to evaluate the scanner configuration in terms of sensitivity and NECR performance. We established a time resolution of 3 ns, and the time coincidence window to 5 ns, mimicking the real scanner. To make the simulation data more realistic, a Gaussian energy blurring of 15% (based on previous experiments performed with these blocks [17]) was included, as well as energy windows of both 30% and 50% (± photopeak position). A paralyzable deadtime of 1 μs was also considered, as estimated from the real DAQ system.

### CASToR reconstruction platform

To reconstruct the list-mode data, we have made use of the CASToR 1.1 platform version [22] and the Siddon projector [23]. CASToR is an open source code that enables to reconstruct not only PET data, but also SPECT and CT as well. CASToR is a generic application that does not estimate correction factors such as normalization, attenuation, scattered, or random counts. Therefore, it is necessary to externally introduce the required correction information.

In particular, we have employed the OSEM algorithm (ordered subsets expectation maximization) with 2 subsets for every reconstruction. The number of iterations has been optimized for the different measurements as it will be described later. 3D images were generated in a binary raw file with a matrix size of 416 × 416 × 50 voxels with 1 × 1 × 1 mm^3^ voxel size. Since monolithic crystals allow one to define the pixel size, somehow based on the measured detector resolution [24], we have selected virtual detector pixels of 1 × 1 mm^2^, which is also a compromise between statistics and computational cost.

### Normalization correction

**Fig. 4:**
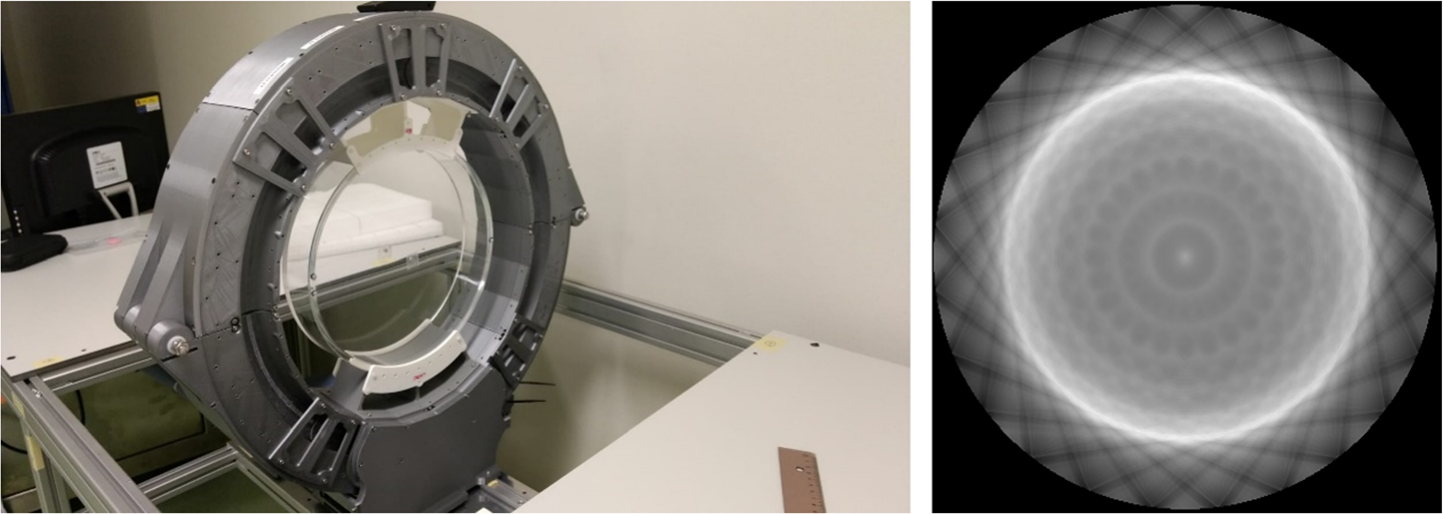
Left, photograph of the detectors ring and normalization phantom. Right, normalization map obtained

Normalization coefficients were calculated across the entire FOV using a custom designed phantom which ensures that all system LORs cross it. The designed phantom consists on a fillable PMMA annulus with inner and outer diameters of 290 mm and 300 mm (50 mm axial), respectively. This particular geometry helps minimizing the number of scattered events [25]. The ring was filled with a solution of FDG and an activity of 7 mCi and positioned in the center of the FOV. Sequential acquisitions over 9 h were carried out. Figure 4 left shows the ProsPET scanner together with the normalization phantom and, on the right, the measured normalization map.

### Attenuation correction

**Fig. 5:**
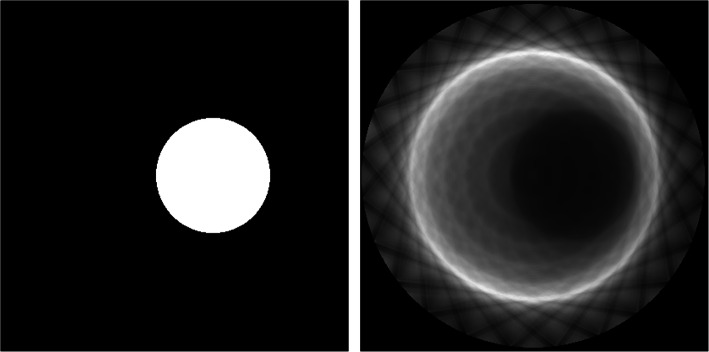
Left, attenuation map of the image quality phantom. Right, combined normalization and attenuation maps

Attenuation maps were either obtained through CT images acquired in a separated system (Philips Gemini-TF 64) or by segmentation in the case of uniform phantoms [26]. In the case of CT-based maps, they were co-registered with the PET acquisitions using a specific software, called ITK-SNAP [27]. We recalculated all voxels to values considering the linear attenuation coefficients of each material. For every data acquisition, we have obtained a correction map that is the combination of the aforementioned normalization and the attenuation one. Figure 5 shows the attenuation and combined attenuation-normalization maps for the image quality phantom (to be defined later). On the left image, all the white values are established to 0.096 cm^−1^, the water value, and the rest are set to 0.

### System spatial resolution and sensitivity

**Fig. 6:**
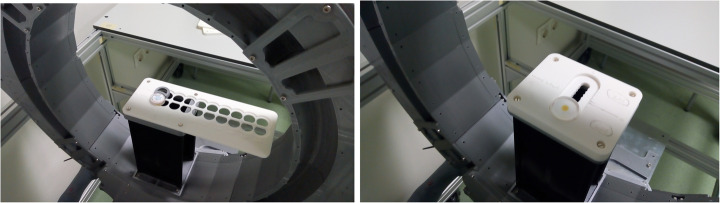
Left, photograph of the holder and source used for the spatial resolution studies along the radial axis. Right, holder for the sensitivity measurements

All tests were performed without including smoothing filters or random and scatter corrections. The spatial resolution is defined as the width of the reconstructed image point spread function (PSF), calculated using the full width at half-maximum (FWHM). Data was acquired using a 0.25 mm in diameter spherical ^22^Na source. This radioactive source had an activity of 22 μCi, and thus, the random coincidence rate is almost negligible, as well as the percentage of dead time losses. Each acquisition lasted 10 min. We designed and constructed holders to place the source in different positions along both the transaxial and axial axes; see Fig. 6. The source was moved along the radial direction in steps of 20 mm, both at the center and at 3/8 of the axial FOV.

The optimal number of iterations for the spatial resolution evaluation was studied using the data acquired when the source was located at the center of the field of view (CFOV). The data was reconstructed both with and without including DOI information.

Using the same radioactive source, we also evaluated the system sensitivity. A new holder that allows one to move the source across the axial direction in steps of 5 mm was designed for this purpose; see Fig. 6. Each measurement lasted 10 min.

### Quality phantom study

We have evaluated the contrast-to-noise ratio (CNR) and the contrast of reconstructed images using a custom-made phantom. The CNR was calculated as follows:

1 $$ \mathrm{CNR}=\frac{\mathrm{Mean}\kern0.5em \mathrm{Hot}\kern0.5em \mathrm{Spot}\kern0.5em \mathrm{VOI}-\mathrm{Background}}{\mathrm{Background}\kern0.5em \mathrm{Standard}\kern0.5em \mathrm{Deviation}} $$

The contrast, in percentage, was calculated using the mean value of each VOI and the background level:

2 $$ \mathrm{Contrast}\left(\%\right)=100\times \frac{\mathrm{Mean}\kern0.5em \mathrm{Hot}\kern0.5em \mathrm{Spot}\kern0.5em \mathrm{VOI}-\mathrm{Background}}{\mathrm{Mean}\kern0.5em \mathrm{Hot}\kern0.5em \mathrm{Spot}\kern0.5em \mathrm{VOI}} $$

The phantom used in this study, named quality phantom (QP), is made out of PMMA and has an outer diameter of 135 mm and 103 mm height. The QP includes 6 insert tubes with different diameters (4.5, 6, 9, 12, 15, 20 mm) and 60 mm height each, at an off-center radius of 35 mm [28]. In addition to filling the inserts with FDG, the background of the phantom was also filled but with a different FDG concentration. Two insert-to-background concentrations were measured, namely 38 and 18. The acquisition of the phantom images lasted 8 min each.

Both the background level and its standard deviation have been calculated. We have defined 12 different volumes of interest (VOIs), with the size of the small insert, distributed along uniform areas of the phantom and obtained the mean value of each one. Thereafter, we generated 6 VOIs that fit each insert dimensions, but with a centered height of 25 mm. The CNR and contrast values as a function of the number of iterations were tested after normalization and attenuation corrections.

For comparison purposes, data acquisitions of the QP using the whole-body PET Gemini-TF 64 (Philips) [29] were carried out about 10 min after the measurements performed with the ProsPET system.

### Count rate performance

**Fig. 7:**
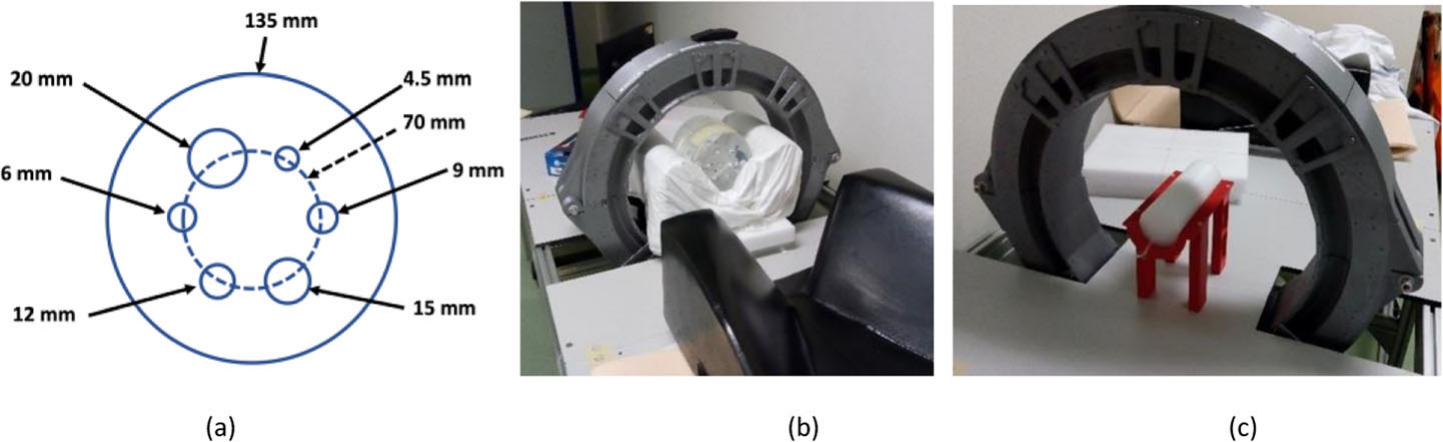
Left, sketch of the QP with positioning of each insert and dimensions (diameters). Center, photograph of the QP while measuring. Right, photograph of the NECR phantom inside the PET ring

In PET imaging, effects such as scattered and random coincidences might generate an image blurring, consequently producing a wrong determination of the radioactive distribution. The intrinsic radiation of LYSO scintillators might also generate undesired random events [30]. Therefore, in order to optimize the quality of the image, it is critical to estimate the percentage of all these losses as a function of the imaged activity. To study the different contributions, we have used a phantom made out of high-density polyethylene with 170 mm length and 60 mm in diameter (see Fig. 7c). It was placed at the scanner center FOV. The phantom has a drilled hole of 3.2 mm in diameter and at 13 mm off-radial direction [24]. Through this hole, a silicone tube (170 mm long) with 1 mm (inner) and 3 mm (outer) diameters was inserted and filled with FDG. The initial activity was 5.45 mCi. We carried out sequential acquisitions every 10 min, during a total time of 18 h.

For each acquisition, a sinogram was generated, where the true, scatter, and random counts are estimated following the NEMA NU 4-2008 protocol [31]. For validation purposes, similar data was obtained using GATE v7.2 simulations.

### Case example: patient study

Finally, we have tested the ProsPET system with a patient that was diagnosed with PCa. The patient was injected with 7.5 mCi of a ^18^F-choline solution. About 90 min post-injection, the patient was scanned in the Philips Gemini-TF (PET/CT) where each bed position lasted 90 s. The CT image was used to determine the distance between the top of the head and the prostate and, therefore, properly positioning the patient within the ProsPET system. The ProsPET acquisition was carried out 30 min after the whole-body PET/CT one. This person signed an informed consent form.

For each of the two axial FOVs we acquired 7 min data. We applied a smoothing post-filter to the final image of 5 mm FWHM transaxial and axial with 3.5 sigmas in the convolution kernel.

## Results

### Detector block performance

After the calibration process, we have determined the average measured detector spatial resolution to be about 1.8 ± 0.4 mm FWHM, combined with an energy resolution of 13.7 ± 1.8 % [17, 18], for all the 24 detectors (see again Fig. 3). A DOI resolution of about 3.2 ± 0.6 mm FWHM for all detectors and for all detection areas allows one to continuously correct to the true LOR [32].

### PET spatial resolution

**Fig. 8:**
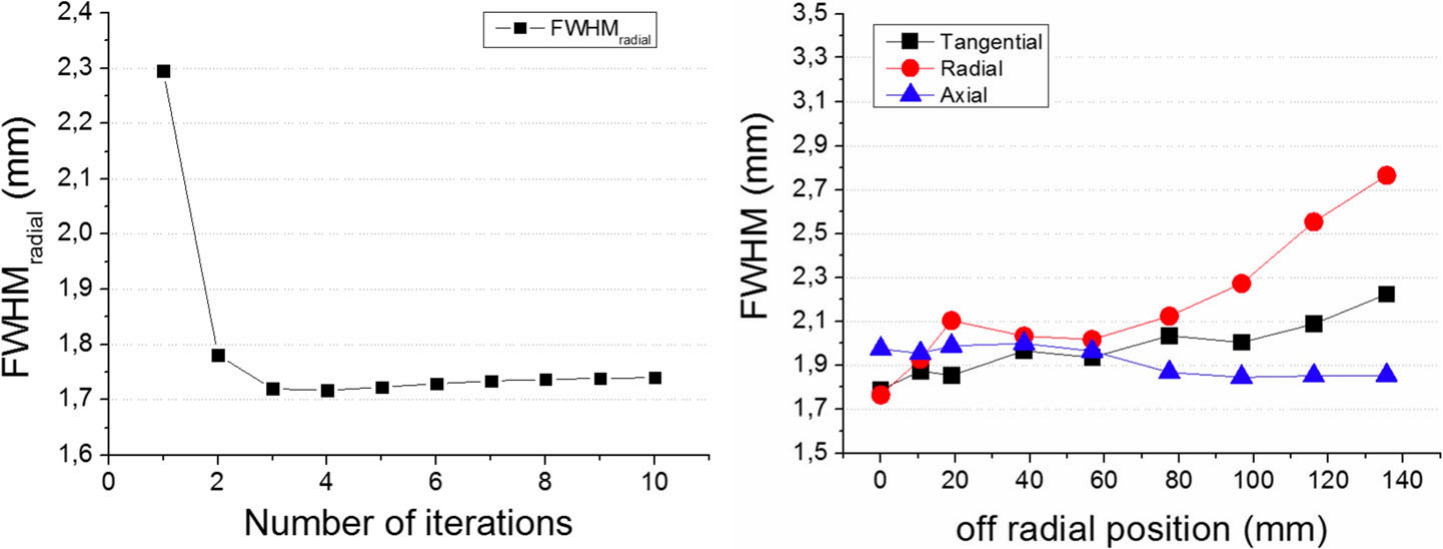
Left, radial FWHM of the source PSF at the CFOV as a function of the number of iterations. Right, tangential, radial, and axial components (FWHM) of the source as a function of the distance to CFOV in radial direction, when using 3 iterations

All acquired data has been corrected for attenuation and normalization, except in the case of the spatial resolution results were only the normalization was applied. Figure 8 left shows the FWHM (radial) of the source at the CFOV as a function of the number of iterations. We observe a convergence of the FWHM at 3 iterations. We used this number of iterations for the reconstruction of the sources along all the other positions in the radial direction. Figure 8 right depicts the measured FWHM for the three space components for the plane *Z* = 0 (axial centered). DOI correction was implemented for these reconstructions. We observe that the three components are below 2 mm FWHM for all positions, except the radial component that increases to about 2.5 mm at the very edge of the FOV.

**Fig. 9:**
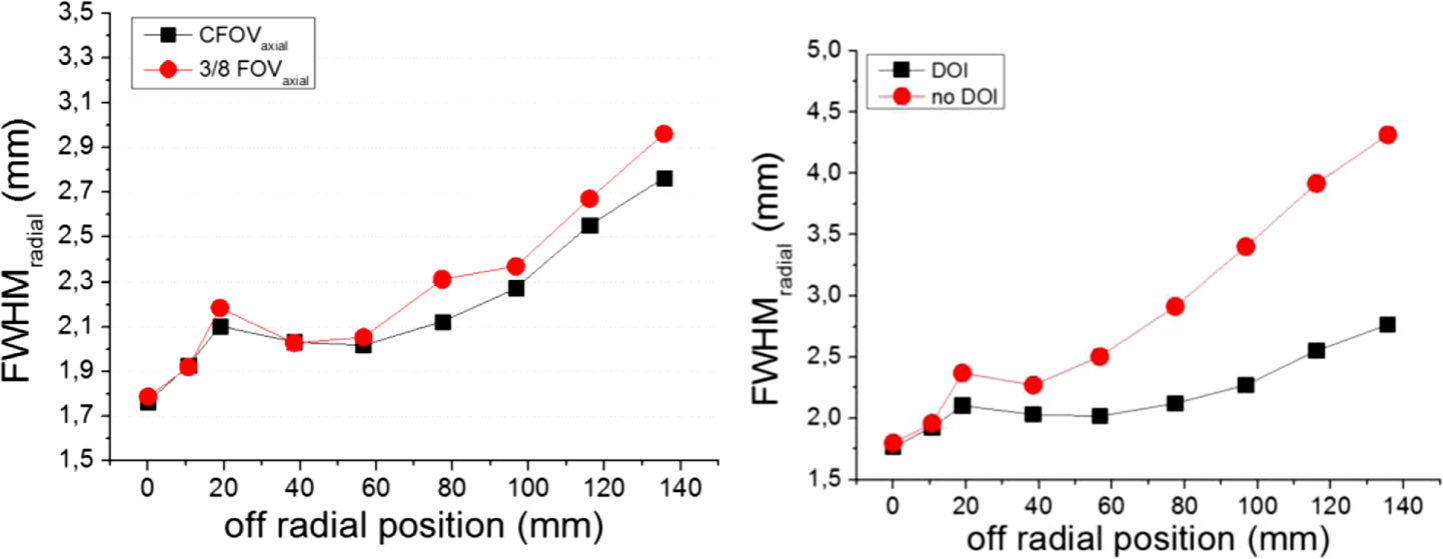
Left, radial FWHM (3 iterations and DOI correction) of the source PSF as a function of the radial position at the axial center and 3/8 (17.25 mm). Right, radial FWHM (3 iterations) as a function of the radial position at the axial center, but with (black squares) and without (red circles) DOI correction

Figure 9 left shows the measured spatial resolution at different radial position when the source is at the center and at 3/8 (17.25 mm) of the axial FOV. As it can be observed, there is almost no degradation in the spatial resolution for the different axial positions. Figure 9 right depicts the radial component with and without DOI correction, making emphasis on the relevance of including 3D photon coordinates information. This radial component increases to as much as 4.5 mm FWHM if DOI is not considered.

### Sensitivity

**Fig. 10:**
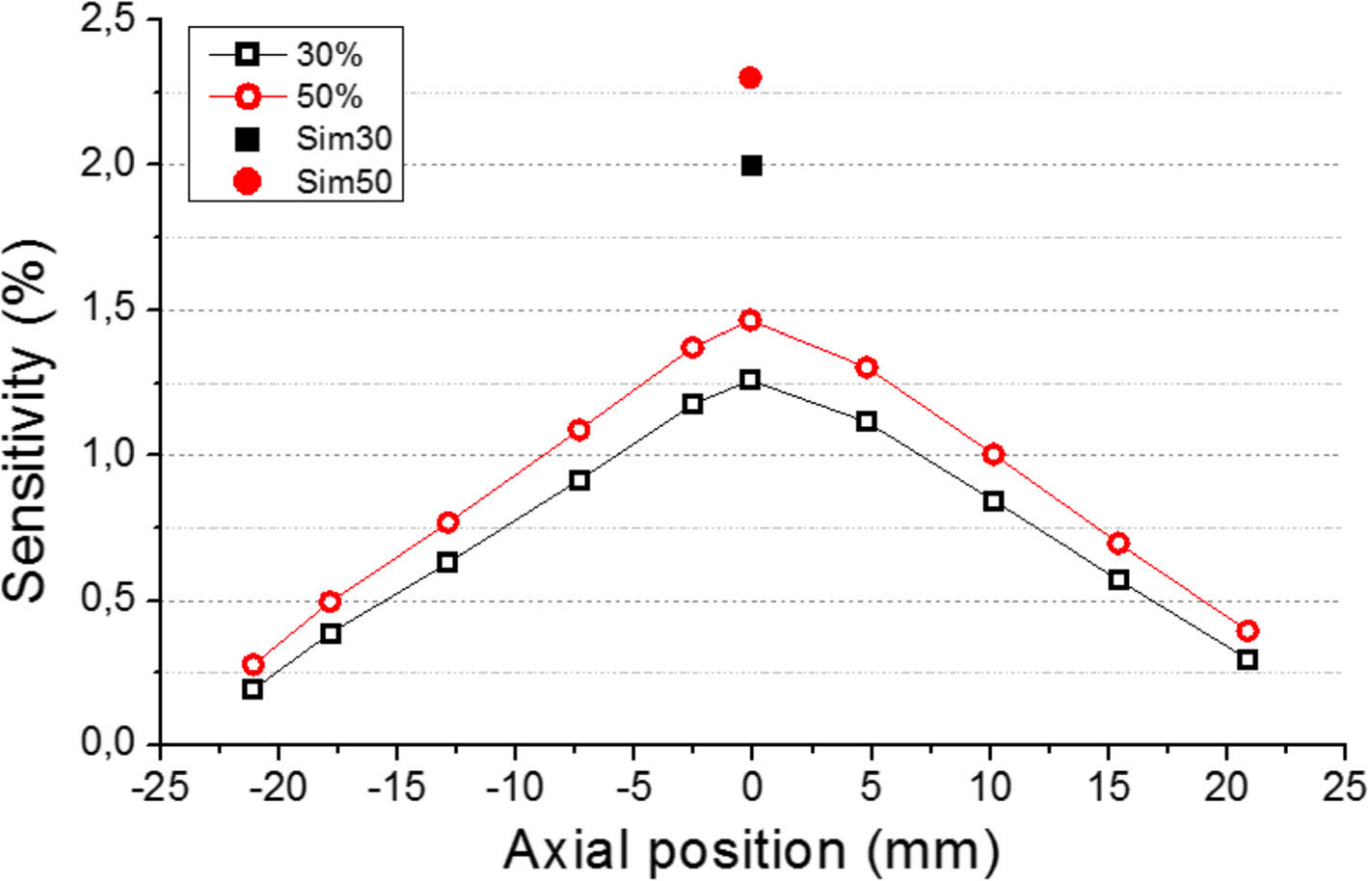
Sensitivity (%) curves for two energy windows 30% (black open squares) and 50% (red open circles), respectively, as a function of the reconstructed source position. The solid black square and red circle exhibit the simulated sensitivity for the 30% and 50% energy windows, respectively

Figure 10 shows the sensitivity profiles as a function of the axial position. As expected, the maximum sensitivity is found at the center of the axial FOV (25 mm in these plots). We have used two different energy windows (EW): 30% (357.7 to 664.3 keV) and 50% (255.5 to 766.5 keV). We have subtracted the background counts. The system achieves 1.46% sensitivity at the scanner center. The sensitivity, estimated by simulations, is also depicted in this plot for both the 30% and 50% EW, respectively.

### Noise-equivalent count rate

**Fig. 11:**
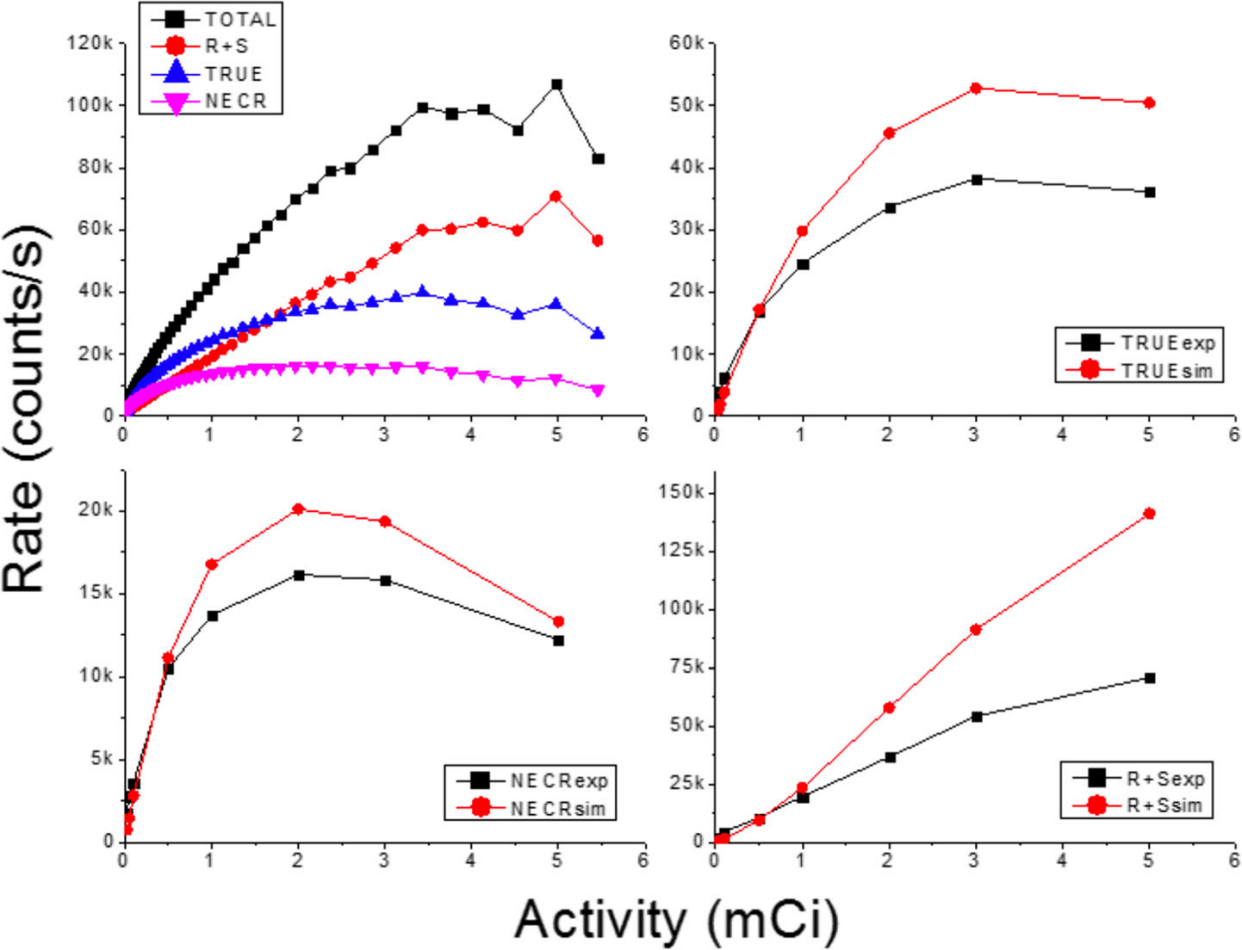
Top-left, measured values for an activity range from 0 to above 5 mCi. Top-right, comparison experimental and simulation data for the true counts. Bottom-left, same as before but for NECR. Bottom-right, same as before but for the sum of random and scatter

Both experimental and simulated data obtained for the NECR phantom were stored in 2D sinograms. After analysis, following the NEMA NU 4-2008 [31], information about the true, random, and scatter counts was obtained as depicted in Fig. 11. The intrinsic radiation of the LYSO scintillation block was not implemented during the simulations but is still present in the experimental data. In order to account for this, we subtracted the measured background counts to the random and scatter contributions.

Figure 11 top-left plots the measured prompt, NECR, scatter plus random, and trues (5 ns CW), as a function of the activity. We observe also in Fig. 11 that both NECR and true counts exhibit similar values for experimental and simulated data, especially at lower activities. However, the curves for the random plus scatter events show some deviations for high activities.

### CNR and contrast results

**Fig. 12:**
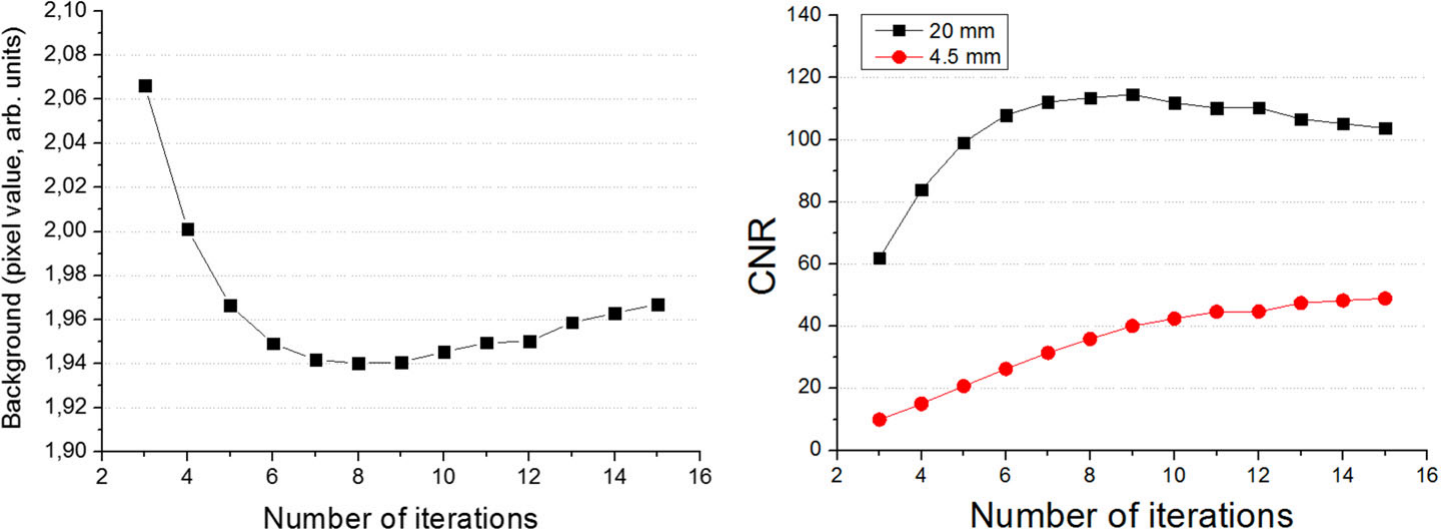
Left, background values as a function of number of iterations. Right, CNR versus number of iterations

Following a similar procedure to the one used during the spatial resolution studies, we have reconstructed the QP using different iterations, as shown in Fig. 12. The reconstructions accounted for normalization and attenuation corrections. Figure 12 right shows the CNR for the smallest and largest inserts within the QP. We have decided to use 8 iterations in the following data analysis, since the background exhibits a minimum at this point.

**Fig. 13:**
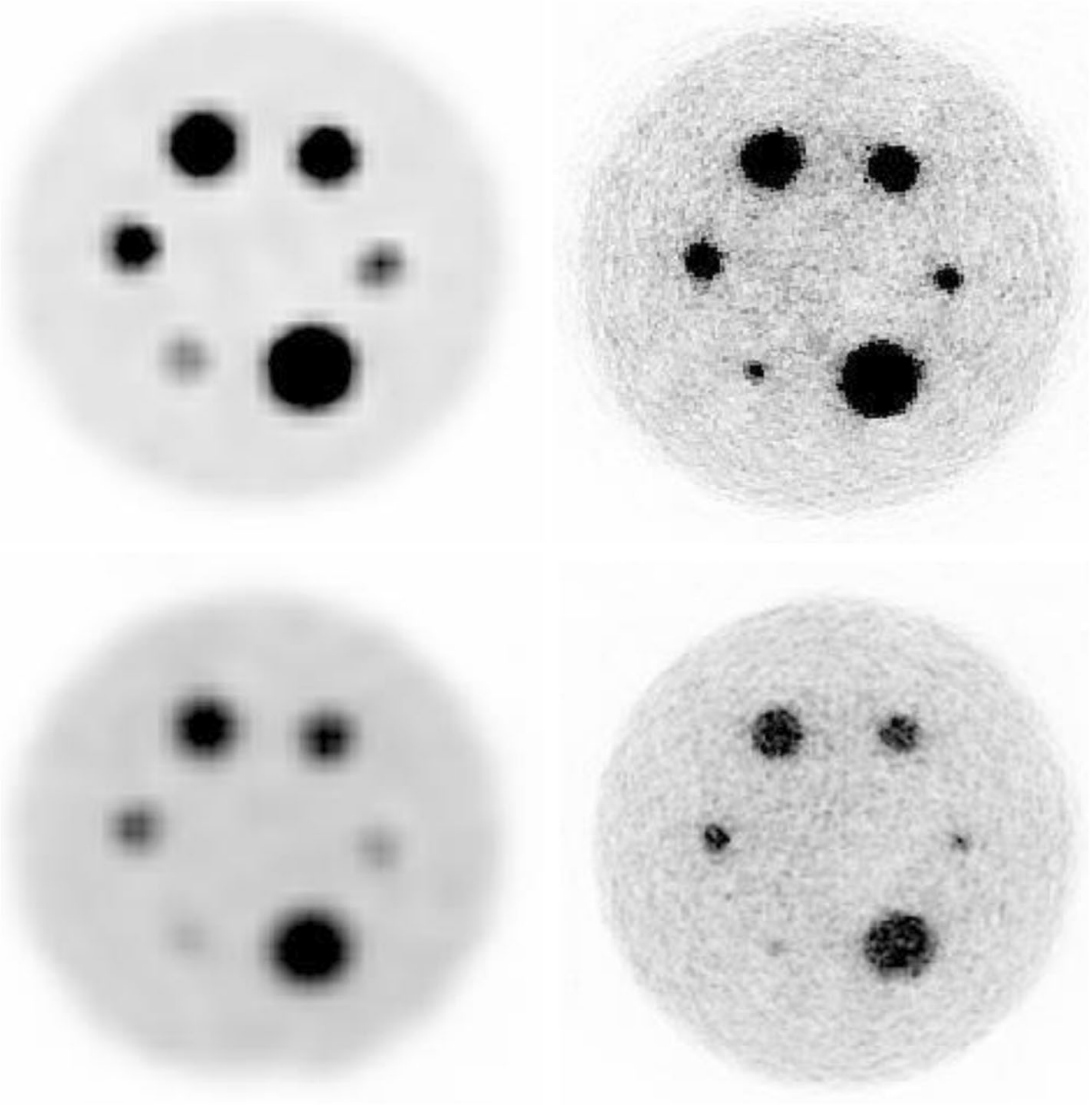
Top, QP with insert-to-background ratio of 38 acquired and reconstructed in the Gemini-TF (left) and with the ProsPET system (1 mm voxel, 8 iterations, attenuation based on CT image, no scatter correction). Bottom, same as top but for an insert-to-background ratio of 18

Figure 13 exhibits the reconstructed QP images. Data acquired with the Gemini-TF was reconstructed using the BLOB-OSEM-TOF algorithm, with 2 iteration and 33 subsets, including single scatter simulation correction and the delayed window approach for the random contribution. No average or smooth filters were applied to any image both for the ProsPET or Gemini-TF.

**Fig. 14:**
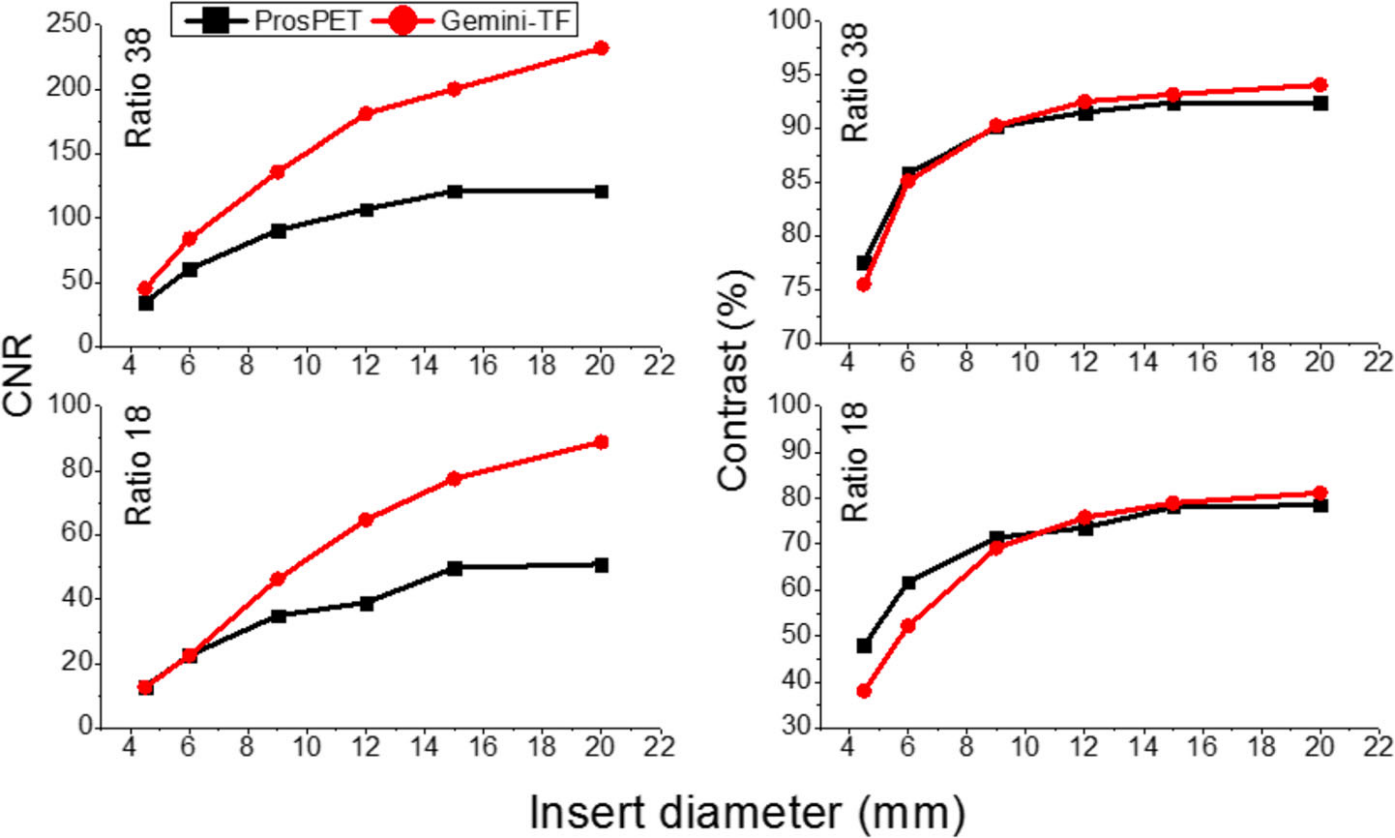
Left, CNR comparison between the ProsPET system using 8 iterations (attenuation based on CT image and no scatter correction) and the Gemini-TF, for the 6 inserts, and the two insert-to-background ratios 18 and 38, respectively. Right, contrast (%) comparison also for all 6 inserts and two ratios

Figure 14 shows the determined CNR and contrast values for both the ProsPET and Gemini-TF reconstructions of the custom QP, for both insert-to-background ratios of 38 and 18. The ProsPET reconstruction used 8 iterations without scatter and random corrections applied, but the attenuation correction based on the CT attenuation map. In terms of the contrast, ProsPET exhibits values higher than 75% for all inserts. Regarding the CNR, although similar values are obtained for both systems for the smallest inserts, differences increase with the size of the inserts, being larger for the whole-body PET.

### Patient study

**Fig. 15:**
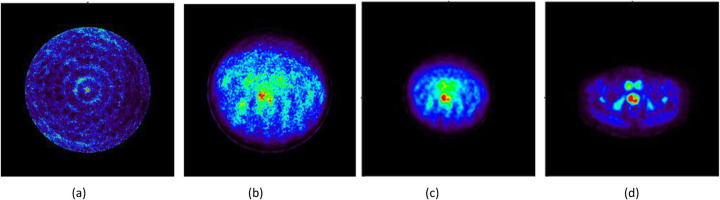
Image reconstructions using the prostate PET prototype of the patient data before normalization (**a**), with only normalization (**b**), also including CT-based attenuation (**c**). Image of one bed position with the Gemini-TF is depicted in **d**

Images obtained with the patient also required normalization and attenuation corrections. We also selected 8 iterations. Figure 15 a and b show the result of the reconstruction if normalization or attenuation was not considered, respectively. We have co-registered the CT and the prostate-dedicated PET images and applied the attenuation map with this information; see Fig. 15c. Figure 15d depicts the image of the same patient acquired with Gemini-TF.

## Discussion and conclusions

The diagnostic image of the primary tumor in prostate cancer is still waiting for his white knight. At present, mpMRI is the imaging modality of choice for precise morphologic evaluation, has higher spatial resolution, and provides clearer anatomic delineation of the prostate and surrounding anatomical structures than PET, while PSMA PET is the superior modality for detecting metastases to the locoregional and extrapelvic lymph nodes, bones, and visceral organs [33]. More than 90% of primary PCa lesions show moderate to high PSMA expression levels on PSMA PET, and many current studies have indicated that PET/MRI could be the single ideal imaging modality for staging PCa patients [33]. Several studies have demonstrated that the intensity of radiotracer accumulation in the primary tumor is correlated to PSA levels and Gleason score and therefore with PCa groups of risk [34, 35]. This is the major cause of our work: considering the potential role of a PSMA PET/US portable device as a prebiopsy diagnostic tool that can be used to guide sampling the most aggressive sites in the prostate, avoiding a high number of samples as in systematic biopsy. Hence, ProsPET could be indicated for detecting intraprostatic malignant lesions in untreated patients with newly diagnosed PCa or oriented to a focal therapy, at an economic cost that makes it accessible to any hospital.

We have designed and built a prostate specific PET system, called ProsPET. This scanner design presents a very small footprint and improved spatial resolution when compared with a conventional whole-body PET scanner. This is caused by the proximity of the detectors to the prostate and more accurate identification of the gamma ray impact within each detector block, including accurate DOI determination. Monolithic scintillator blocks are being used in this design, which combined with our readout electronics and data processing techniques offer the capabilities to accurately determine the 3D gamma ray interaction coordinates within the scintillator block and, thus, to correct the parallax error. When this correction is not enabled in the reconstructed data, the spatial resolution at the edges of the FOV worsens; see Fig. 9.

As in most clinical PET systems, there is a need for normalization and attenuation corrections. This becomes more important for regions such as the thorax or the hip where multiple organs and surrounding bones are present. We have calculated a generic normalization map for every measurement and a specific attenuation map for each experiment.

The ProsPET prototype exhibits a comparable sensitivity (1.5% at the FOV center) than conventional PET systems. The Gemini-TF reaches 0.7% at the FOV center [29]. We have observed some differences between the sensitivity values obtained with the simulations and with experimental data, most likely caused by losses in the real data not considered in the simulations. In terms of spatial resolution, the FWHM of the sources at the FOV center is around 2 mm while the Gemini-TF reports around 4.7 mm [29]. With the DOI correction enabled in the ProsPET, the spatial resolution is always below 3 mm for the entire FOV.

One limitation that we have observed studying the count rate curves is that ProsPET shows a high contribution of random and scatter events, even for low activities. We are currently working on implementing these two contributions. We found the NECR maximum point at 16 kcps for an activity of 2.4 mCi. The high contributions of random and scatter events affect the CNR but not the image contrast. The differences between real and simulated data at high activities might also be caused by an underestimation of the simulated electronics dead time.

The SNR exhibits certain differences for the larger inserts of the QP, most likely, as a consequence of the high random and scatter contribution, observed in the NECR rates. However, due to the high spatial resolution of the ProsPET, this shows a higher contrast for the smaller insert diameters, which becomes comparable for largest inserts.

Finally, we have tested the ProsPET with a patient with prostate cancer. Multiple lesions were detected both using the whole-body PET but also the ProsPET. Although the result was positive and the lesions detected, the dedicated system still requires some further improvement when correcting the patient attenuation. This was easily achieved with phantoms but a better co-registration is needed when using patients. Regarding comfortability, patients felt comfortable within the dedicated PET system.

## Data Availability

The datasets used and/or analyzed during the current study are available from the corresponding author on reasonable request.
